# Progress of cGAS-STING signaling in response to SARS-CoV-2 infection

**DOI:** 10.3389/fimmu.2022.1010911

**Published:** 2022-12-07

**Authors:** Yaru Wu, Min Zhang, Cui Yuan, Zhenling Ma, Wenqing Li, Yanyan Zhang, Lijuan Su, Jun Xu, Wei Liu

**Affiliations:** ^1^ College of Life Sciences, Henan Agricultural University, Zhengzhou, China; ^2^ College of Pharmacy, Henan University of Chinese Medicine, Zhengzhou, China

**Keywords:** SARS-CoV-2, coronavirus, innate immunity, STING, vaccine adjuvants

## Abstract

Coronavirus disease 2019 (COVID-19) is an epidemic respiratory disease caused by severe acute respiratory syndrome coronavirus 2 (SARS-CoV-2) that can cause infections in millions of individuals, who can develop lung injury, organ failure, and subsequent death. As the first line of host defense, the innate immune system is involved in initiating the immune response to SARS-CoV-2 infection and the hyperinflammatory phenotype of COVID-19. However, the interplay between SARS-CoV-2 and host innate immunity is not yet well understood. It had become known that the cGAS-STING pathway is involved in the detection of cytosolic DNA, which elicits an innate immune response involving a robust type I interferon response against viral and bacterial infections. Nevertheless, several lines of evidence indicate that SARS-CoV-2, a single-stranded positive-sense RNA virus, triggered the cGAS-STING signaling pathway. Therefore, understanding the molecular and cellular details of cGAS-STING signaling upon SARS-CoV-2 infection is of considerable biomedical importance. In this review, we discuss the role of cGAS-STING signaling in SARS-CoV-2 infection and summarize the potential therapeutics of STING agonists as virus vaccine adjuvants.

## Introduction

Every outbreak of coronaviruses (CoVs) with novel viral antigens has caused severe economic and societal damage. As early as 2003, severe acute respiratory syndrome coronavirus (SARS-CoV) caused the first major pandemic disease of the new millennium (1). In 2012, the outbreak of a new pathogenic coronavirus, the Middle East Respiratory Syndrome Coronavirus (MERS-CoV), occurred in the Arabian Peninsula (2). In 2019, SARS-CoV-2 caused an infectious disease pandemic worldwide (3). To date, SARS-CoV-2 has infected hundreds of millions of people, and its infection continues (4). Common symptoms of infected individuals include respiratory symptoms, fatigue, fever, cough, hypogeusia/hyposmia, shortness of breath, and dyspnea (5). Furthermore, infection can cause pneumonia, severe dyspnea, and even death (6). However, there is currently no effective and specific treatment for diseases caused by SARS-CoV-2 (7).

To date, the genus CoVs can be subdivided into four clusters based on genetic and serologic properties, including Alphacoronavirus (α), Betacoronavirus (β), Gammacoronavirus (γ), and Deltacoronavirus (δ) (8). SARS-CoV-2 has a broad host range, including humans, bats, hamsters, ferrets, and other mammals (9, 10). It has been reported that infected animals can infect humans, leading to sustained human-to-human transmission (11). SARS-CoV-2 is classified as the genus Betacoronavirus, which has a positive-sense single-stranded RNA (+ssRNA) genome of ~30 kb (12). Its genome contains two large open reading frames (ORFs), *ORF1a* and *ORF1ab*, which are proteolytically cleaved by main protease (Mpro, 3CLpro, or nsp5) and papain-like protease (PLpro) to form 16 nonstructural proteins (nsp1-16), four structural proteins and eleven accessory proteins. Among them, the nsp1-16 participate in genome replication and early transcription regulation (13). The structural proteins include membrane (M), nucleocapsid (N), envelope (E), and spike (S), which are common to all coronaviruses (14). Accessory proteins of SARS-CoV-2 consist of ORF3a, ORF3b, ORF3c, ORF3d, ORF6, ORF7a, ORF7b, ORF8, ORF9b, ORF9c and ORF10 (15). Based on previous research, efficient replication and population transmission of SARS-CoV-2 suggests that the virus can effectively circumvent the human innate immune responses (16). In particular, SARS-CoV-2 lineages have diverged into highly prevalent variants, such as Delta (B.1.617.2), which are easier to spread and potentially aggravate the severity of respiratory diseases and reduce the effectiveness of COVID-19 vaccines (17). Therefore, it is essential to reveal the roles of SARS-CoV-2 in innate immunity. The innate immune system is the first line of immunological defense for rapidly eliminating viruses. Pattern recognition receptors (PRRs) are key parts of the innate immune system against viruses, as they detect viral pathogen-associated molecular patterns (PAMPs) (18). A significant factor of host response to viral infections largely depends on the initial activation of PRRs by viruses, primarily by viral DNA or RNA (19). The signaling pathways activated by PRRs result in the expression of proinflammatory cytokines to recruit immune cells and type I as well as type III interferons (IFNs), thereby inducing interferon-stimulated genes (ISGs), which are powerful virus restriction factors to establish the antiviral state (20). Cyclic GMP-AMP synthase (cGAS) is a cytosolic PRR that can recognize cytosolic nucleic acids, including dsDNA (21). The recognition of dsDNA by cGAS produces cyclic GMP-AMP (cGAMP). cGAMP is subsequently recognized by stimulator of interferon genes (STING, also nominated as MITA/MYPS/ERIS), which exits the endoplasmic reticulum (ER) and translocates to the Golgi, where STING triggers type I IFNs and proinflammatory responses (22–24). In the absence of viral infection, cGAS-STING signaling plays an important role in autoimmune diseases, antitumor activity, cancer immunotherapy, tissue disorder regulation, autoinflammation and so on (25). Upon viral infection, STING also acts as a scaffold protein for TANK-binding kinase 1 (TBK1) and IRF3 and links them to the MAVS complex in mitochondria. Moreover, STING activation is critical for the stimulation of IRF3 activity (26). Hence, upon activation, STING forms dimers that assemble with MAVS, TBK1 and IKKϵ, leading to IRF3 activation and subsequent induction of IFNs. In addition, STING activation also leads to the induction of autophagy responses, resulting in a strong inhibitory effect against infections which are caused by a variety of microbial pathogens (27). Liu et al. found that STING contains LC3 interacting regions (LIRs) and mediates autophagy through direct interaction with LC3 (28).

Furthermore, the cGAS-STING pathway is basically induced by leaked mitochondrial DNA, microbial DNA, extranuclear chromatin, cytosolic micronuclei, and aberrant chromosomal DNA (29, 30). Thus, recognition of cytosolic dsDNA mediated by cGAS-STING signaling leads to the induction of type I interferon, which plays an important role in innate immunity against cytosolic pathogens. Zhou et al. reported that SARS-CoV-2 facilitates intercellular fusion *via* the S protein, resulting in the shuttling of chromatin DNA from the nucleus and eventually activating the cGAS-STING pathway (31). However, Lv and others speculated that SARS-CoV-2 may cause mitochondrial damage, leading to a build-up of mtDNA in the cytoplasm (32). Then, the cGAS-STING pathway is activated by raised mtDNA leakage. It was interesting to determine how SARS-CoV-2, a single-stranded positive-sense RNA virus, triggered the cGAS-STING signaling pathway. Nevertheless, the role of the cGAS-STING pathway triggered by SARS-CoV-2 is still unknown. This review provides insights into the response of cGAS-STING signaling pathways to SARS-CoV-2 infection and suggests potential therapeutic drugs for SARS-CoV-2. The therapeutic targeting of cGAS-STING signaling may provide a novel approach to treat autoimmune and inflammatory diseases.

## cGAS-STING pathways induced by SARS-CoV-2

**Figure 1:**
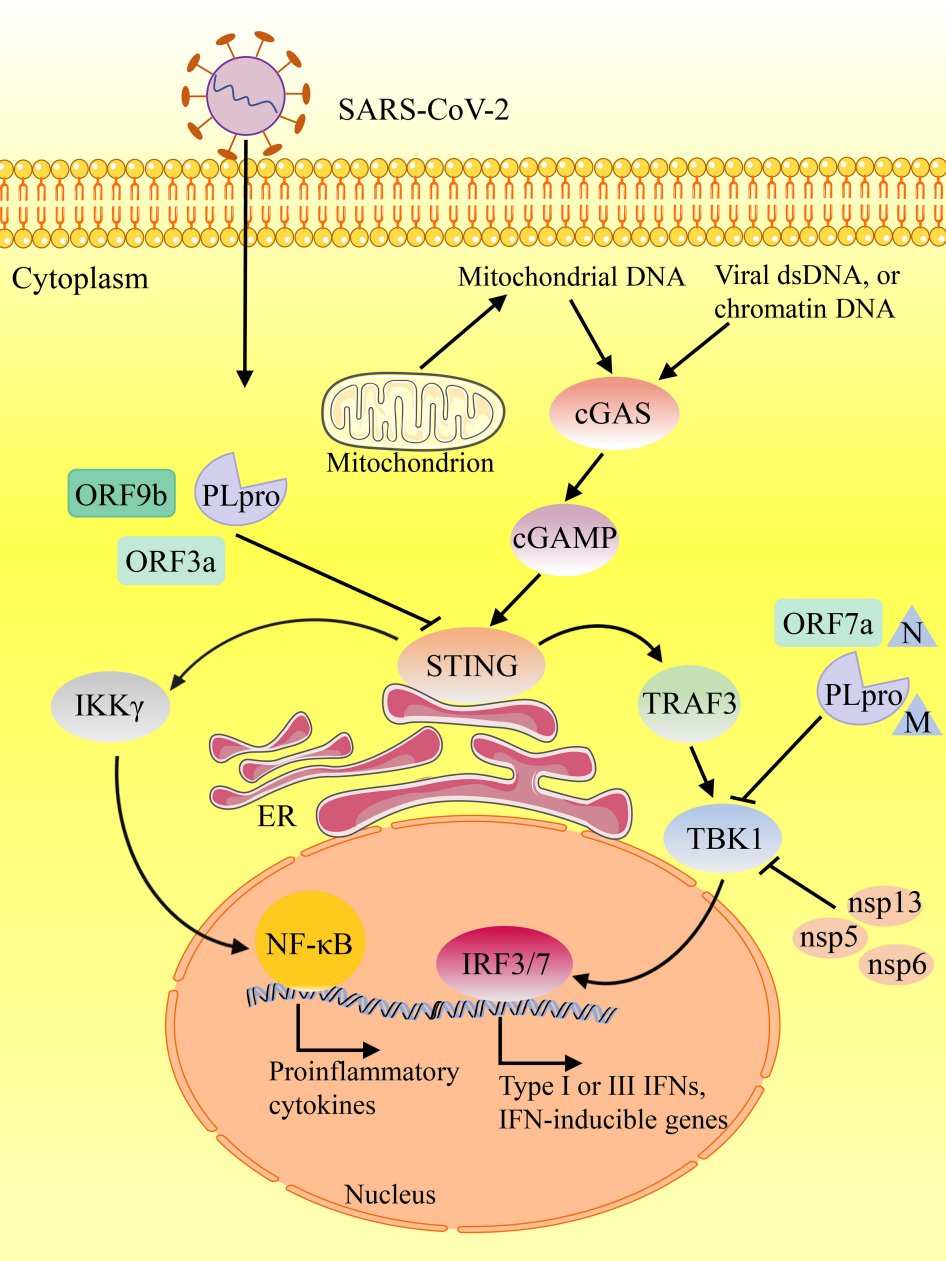
The contributing role of STING in innate responses against SARS-CoV-2 infection. SARS-CoV-2 suppresses the components of cGAS-STING pathway, resulting in inhibiting the synthesis and release of various proinflammatory cytokines and type I IFNs.

Multiple lines of evidence have indicated that the cytoplasmic DNA sensor cGAS-STING recognizes dsDNA viruses, but also plays a critical role in RNA virus infection, either by directly recognizing characteristics of virus or by detecting cellular DNA released from mitochondria or nuclei in responding to cellular stress (33). Mdkhana et al. reported that STING could play an important role in SARS-CoV-2 infection by inducing the type I IFN response (**Figure 1**) (32, 34, 35). Although rapid induction of type I IFNs limits virus propagation, a sustained increase in the levels of type I IFNs in the late phase of the infection is associated with aberrant inflammation and poor clinical outcome (36). Evidence shows that the cGAS-STING pathway is a critical determinants of aberrant type I IFN responses in COVID-19 (37). Application of STING inhibitor suppressed STING activation, thereby reducing severe lung inflammation induced by SARS-CoV-2 and improves disease outcome (32, 36). cGAS-STING activation leads to increased inflammation and pathogenesis in infected patients and mice in COVID-19 (36). Neufeldt et al. found that SARS-CoV-2 infection activates the cGAS-STING pathway, which leads to induction of proinflammatory cytokines mediated *via* nuclear factor κB (NF-κB) pathway and this response can be controlled with STING inhibitors (33). Neufeldt et al. observed colocalization between STING and SARS-CoV-2 N protein in infected cells, and Rui Y et al. showed interactions between STING and ORF3a, both suggesting viral proteins play a direct role in manipulating the cGAS-STING pathway (33, 38).

### Current understanding of SARS-CoV-2 ORFs in cGAS-STING pathways

Host responses to viral infection are dependent on mitochondrial functions (39). Manipulation of host mitochondria by ORFs of SARS-CoV-2 can lead to leakage of mtDNA into the cytoplasm, activate mtDNA-triggered inflammasomes and inhibit innate and adaptive immunity (40). Indeed, mtDNA is a proinflammatory damage associated molecular pattern (DAMP) and has a pathogenic role in a variety of inflammatory diseases (41). SARS-CoV open reading frame-9b (ORF9b) localizes to the mitochondrial membrane (42). Viral genome mutations commonly exist, but mutation coldspots may serve as conservative diagnostic and therapeutic targets in ORF9b, and no mutations were detected in ORF9b from publicly available data (43). SARS-CoV ORF9b is a unique accessory protein with a novel fold that can form a dimeric tent-like β structure with an amphipathic surface and a central hydrophobic cavity which binds lipid molecules encoded by an another ORF within the N gene (44).

Gao et al. reported that SARS-CoV-2 ORF9b inhibits type I IFN response through its central portion of ORF9b occupying the deep pocket in the C-terminal domain (CTD) of TOM70, which is a 70-kDa membrane-anchored adapter that relays antiviral signaling from MAVS to TBK1/interferon regulatory factor 3 (IRF3) (45). In addition, ORF9b inhibits RIG-I-MAVS antiviral signaling through its N-terminus by interrupting K63-linked ubiquitination of IKKγ, which is the NF-κB essential modulator (46). Once the virus enters the host cell, ORF9b can directly manipulate mitochondrial function to escape host cell immunity and promote virus replication and COVID-19 disease. Furthermore, SARS-CoV-2 ORF9b impairs the induction of type I and III IFNs by targeting several components of the cGAS-STING signaling pathways (47). Confocal microscopy analysis reveals that SARS‐CoV‐2 ORF9b colocalized with mitochondria, whereas partially localized to the ER *via* interaction with STING (47). The transfection of the SARS‐CoV‐2 ORF9b plasmid significantly decreased the phosphorylation of TBK1, which phosphorylates IRF3, and this phosphorylation results in the translocation of IRF3 into the nucleus to induce the production of types I and III IFNs and other proinflammatory cytokines (47). Similarly, SARS‐CoV‐2 nsp5, nsp6, nsp13 and N protein were reported to inhibit the phosphorylation of TBK1 (48–52). Nevertheless, SARS-CoV-2 M protein induces TBK1 degradation through K48-linked ubiquitination, thereby significantly inhibiting the production of IFNs (53–56). Interestingly, host factor USP22 can also regulate human intestinal epithelial cells (hIECs) to secrete type III IFNs and SARS‐CoV‐2 infection. Karlowitz et al. found that USP22 controls basal and 2’3’-cGAMP-induced STING activation, and serves as central regulator of basal IFN-λ secretion (57). USP22-deficient human intestinal epithelial cells are protected against SARS-CoV-2 infection, viral replication, and the formation of virus particles, in a STING-dependent manner. Likely to ORF9b, ORF10 targets STING to antagonize IFN activation (58). Overexpression of ORF10 inhibits cGAS-STING induced IRF3 phosphorylation, translocation, and subsequent IFN induction. Moreover, ORF10 also prevents the ER-to-Golgi trafficking of STING by anchoring STING in the ER. Therefore, these findings suggested that SARS‐CoV‐2 ORFs acts as a negative regulator of antiviral immunity, thereby modulating the cGAS-STING pathway.

Open reading frame-3a (ORF3a), a highly conserved coronavirus protein, is the largest accessory protein of SARS-CoV-2, which locates on the plasma membrane and endomembranes, including lysosomes, endosomes, Golgi, and endoplasmic reticulum (ER), and disturbs with ion channel activities in host plasma and endomembranes (59). ORF3a is involved in virus replication and release, autophagy blockade, proapoptotic activity, promotion of lysosomal exocytosis, and inflammasome activation (15, 60, 61). Rui et al. reported that ORF3a mediated the activation of cGAS-STING function and possessed a certain inhibitory effect on nuclear accumulation of NF-κB p65 (38). Furthermore, ORF3a could interact independently with the N-terminal and the C-terminal fragment of STING, a truncated STING mutant lacking both the N- and C-terminal regions failed to interact with ORF3a, indicating that ORF3a participates in regulating the production of type I and III IFNs (38). In addition, ORF3a was capable of suppressing the activity of STING activated by STING agonists (38). Notably, SARS-CoV-2 ORF3a can also inhibit STING function in human, mouse, and chicken (38). Thus, ORF3a-mediated inhibition of cGAS-STING may be due to the inhibition of STING, rather than cGAS. However, Su et al. discovered that SARS-CoV-2 ORF3a can interact with STING and disrupt the STING-LC3 interaction, thus effectively blocking cGAS-STING-induced autophagy but not IRF3-type I IFN induction (62). STING contains LC3 interacting regions and mediates autophagy through direct interaction with LC3 (28).

### Proteases of SARS-CoV-2 in cGAS-STING pathways

Similar to ORF3a, 3CLpro-mediated suppression of cGAS-STING may be due to the inhibition of STING rather than cGAS, and suppresses cGAS-STING-mediated p65 nuclear accumulation (38). 3CLpro forms a dimer with two monomers (residues 1-306), and each one has three domains (domains I, II and III) (63). Although the monomeric state of 3CLpro is inactive, the homodimeric state formed by the dimerization of two monomers is active (64). Rui et al. found that 3CLpro possessed the ability to suppress immune responses induced by both the RLR and cGAS-STING pathways (38, 65). The ability of cGAS-STING to activate NF-κB signaling was inhibited more effectively by 3CLpro than was that triggered by downstream factors in the cGAS-STING pathway such as IKKα, IKKβ, TBK1, p65 and IKKϵ (38). Immunoprecipitation indicated that 3CLpro bound to STING and specifically inhibited the K63-linked ubiquitylation of STING to impair the STING functional complex assembly and downstream signaling (38). Similar to ORF3a, various vertebrate STINGs, involving humans, mice, and chickens, could be suppressed by 3CLpro of SARS-CoV-2 (38). These findings show that SARS-CoV-2 3CLpro may target the conserved features of vertebrate STING molecules. Another protease of SARS-CoV-2, PLpro, is a potent inhibitor of cGAS-STING function, which could significantly reduce K63-ubiquitination of TBK1 for suppressing IFNs production and signaling (66).

### Spike protein of SARS-CoV-2 in cGAS-STING pathways

The spike (S) protein of SARS-CoV-2 is composed of two subunits, S1 and S2, which plays a key role in the receptor recognition and cell membrane fusion process. Several lines of evidence have indicated that SARS-CoV-2 facilitates intercellular fusion *via* the spike (S) protein. Ren et al. reported that viral infection could induce syncytia formation within cells expressing ACE2 and the SARS-CoV-2 S protein, leading to the production of micronuclei (67). cGAS recognizes chromatin DNA shuttled from the nucleus resulting from intercellular fusion upon SARS-CoV-2 infection, which results in a high level of activation of cGAS-STING signaling and induces a type I interferon (IFN) response (31, 68). Furthermore, cytoplasmic chromatin-cGAS-STING pathway, contributes to IFN and pro-inflammatory gene expression upon cell fusion (31). However, phosphorylation of the transcriptional regulator IRF3 and the encoding of type I IFN, were reduced in cGAS-deficient fused cells (68). Therefore, these results suggested that the potential role of cGAS in cell-cell fusion from SARS-CoV-2.

## Therapeutic drugs targeting STING

A previous study provided evidence that STING activation represents a prospective therapeutic treatment to control SARS-CoV-2 (**Table 1**) (69). Several recent studies have shown that STING agonists interfere in SARS-CoV-2 infection by regulating the type I IFN response (69, 75). In order to reveal antiviral innate immune agonists to block SARS-CoV-2 infection, Li et al. conducted high-throughput screening and identified endogenous STING agonists, cyclic dinucleotides (CDNs), as antiviral agents against SARS-CoV-2 (69). Due to the low potency of CDNs and poor drug quality, potent small molecule STING agonists have been exploited, such as diABZI (76–78). Li et al. examined the small molecule STING agonist diABZI and detected that it can effectively inhibit SARS-CoV-2 infection of various strains, including the South African variant B.1.351, through transiently stimulating IFN signaling (69). Notably, diABZI can impair viral replication in primary human bronchial epithelial cells and in mice *in vivo*. Therefore, this STING agonist could be utilized as a new therapeutic strategy against COVID-19. Similarly, Humphries et al. described a diamidobenzimidazole compound, diABZI-4, which activates STING and is highly potent in restricting SARS-CoV-2 replication in cells and animals (70). diABZI-4 blocked SARS-CoV-2 replication in lung epithelial cells. Intranasal delivery of diABZI-4 leaded to a rapid short-lived activation of STING, contributing to transient proinflammatory cytokine production and lymphocyte activation in the lung, which is related to suppression of viral replication (70). Hence, diABZI-4 possesses a wide range of protective effects against respiratory SARS-CoV-2 infections. In addition, it was found that there are several new cGAS-STING activators, such as colloidal manganese salt (Mn jelly, Mn J), CF501, mucoadhesive nanoparticles, and IAPA (indirect-acting pan-antiviral) agents, which provide new ideas for anti-SARS-CoV-2 therapy (71–74).

**Table 1:** Potential therapeutic strategy for targeting STING in SARS-CoV-2.

Name	Therapeutic strategy	Description
diABZI	STING agonist	Significantly suppresses SARS-CoV-2 infection (69);
diABZI-4	STING agonist	Stimulates STING and is highly effective in inhibiting SARS-CoV-2 replication (70);
CF501	STING agonist	Vaccine to resist the current epidemic SARS-CoV-2 and its variants (71);
mucoadhesive nanoparticles	STING agonist	The intranasal delivery system loaded with cGAMP potently boosted the immunogenicity of the spike vaccine in the respiratory tract (72);
IAPA agents	STING agonist	Boosts the immune system through STING while blocking essential inflammation-related proteins such as caspase-1 and TNF-α (73);
Mn J	STING agonist	Mn J was made to serve not only as an immune enhancer but also as a delivery system to activate humoral and cellular immune responses (74);

To prevent infection, disease, or transmission of SARS-CoV-2, effective vaccines are considered essential to reduce further morbidity and mortality (79). For nearly a century, Aluminum-containing adjuvants have been utilized to boost immune responses in the billions of doses of vaccines. Along with the evolution of the biocompatible platforms of peptide, protein and biomembrane, the stability of STING agonists has been improved (80). So far, only a few adjuvants have been approved for humans use, of which aluminum-containing adjuvant is the only one widely used (81). However, the nanoparticle manganese (nanoMn) adjuvant (also known as STING agonists) have been shown to promote antigen presentation, virus-specific memory T-cell development and host-adaptive immunity, making it an optimum adjuvant for protein-based COVID-19 subunit vaccines (**Table 2**) (84). Zhang et al. reported that the nanoMn adjuvant is the most effective in strengthening immunogenicity or immune responses of SARS-CoV-2 protein-based subunit vaccines (86). Moreover, a novel STING agonist, CDG^SF^, as an adjuvant immunization with SARS-CoV-2 S protein, causes extremely high antibody titers and a strong T-cell response, overcoming the shortcomings of aluminum-containing adjuvants (85). In addition, the vaccine-stimulating immune response of the respiratory tract is significant in controlling SARS-CoV-2 transmission and disease development (87). Mucoadhesive nanoparticles were utilized to convey SARS-CoV-2 S protein into the nasal tracts of mice (72). Moreover, An et al. reported that NanoSTING as the adjuvant for intranasal vaccination of S protein trimeric or monomeric form elicited robust serum neutralizing antibodies and T-cell responses (82). The administration of the S protein together with cGAMP resulted in a strong stimulatory effects on antibody responses in the respiratory tract (88, 89). Moreover, the induced antibodies can neutralize the wild-type and Delta variant strains of SARS-CoV-2 (72). The data showed that STING agonists had a strong adjuvant effect on the immunogenicity of the S protein (74). In general, these findings underlined the adjuvant potential of the STING agonist in the SARS-CoV-2 vaccine. STING can be activated rapidly and transiently by STING agonists, which stimulate IFNs signaling instantaneously (69, 70). Nevertheless, for STING vaccine adjuvants that cause durable humoral and cellular immune responses, it is noteworthy to avoid the deterioration of the disease caused by excessive inflammation (71).

**Table 2:** Potential vaccine adjuvants targeting STING of SARS-CoV-2.

Name	Therapeutic strategy	Description
NanoSTING	vaccine adjuvant	NanoSTING as the adjuvant for intranasal vaccination of S protein trimeric or monomeric form (82);
cGAMP-ternary adjuvant system	vaccine adjuvant	A novel ternary adjuvant system with Alum/STING agonist 3,3’-cGAMP/poly(I:C) (83);
nanoMn	vaccine adjuvant	Enhances cellular uptake and sustained release of Mn^2+^ in a pH-sensitive manner, thereby enhancing IFN response (84);
CDG^SF^	vaccine adjuvant	CDG^SF^ as an adjuvant immunization with SARS-CoV-2 S protein (85);

STING, stimulator of interferon genes; SARS-CoV-2, severe acute respiratory syndrome coronavirus 2; cGAMP, cyclic GMP-AMP; IAPA, indirect-acting pan-antiviral; TNF-α, tumor necrosis factor-alpha; poly(I:C), TLR3 agonist; nanoMn, nanoparticle manganese; IFN, interferon; Mn J, colloidal manganese salt; S protein, spike protein.

Aberrant activation of STING could exacerbate inflammatory disease that has been linked to severe COVID-19. Similarly, severe disease symptoms induced by SARS-CoV-2 are often associated with high levels of pro-inflammatory cytokines, IFNs and low antiviral responses, which can cause systemic complications. Neufeldt et al. reported that SARS-CoV-2 directs a cGAS-STING mediated, NF-κB-driven inflammatory immune response in human epithelial cells that likely contributes to inflammatory responses seen in patients (33). In addition, Lv et al. found that cGAS-STING mediated immune dysregulation is associated with age (32). Macrophages with shortened telomeres, exhibited hallmarks of cellular senescence, mitochondrial distress, abnormal activation of STING, which predisposed mice to severe viral pneumonia during commonly mild infections. Therefore, limiting STING activation leads to decrease inflammatory responses and decreased pathogenesis, suggesting that STING can be used as a therapeutic target to inhibit severe disease symptoms caused by SARS-CoV-2.

## Discussion

The roles of the STING signaling pathway in the process of SARS CoV-2 infection have been reported. It is understood that SARS-CoV-2 ORF9a or ORF3 damaged type I and III IFNs by targeting several constituents of the cGAS-STING signaling pathways, thus promoting virus replication. On the other hand, protease of SARS-CoV-2, 3CLpro can inhibit STING and destroy the assembly of the STING complex and downstream signal transduction. Recently, it was reported that the upstream signal molecule of STING, cGAMP, which is an immune-stimulating molecule produced by cGAS, can be used as an adjuvant of the COVID-19 vaccine. cGAMP loading of virus-like particles (VLPs) containing the SARS-CoV-2 S protein increases S-specific antibody titers. Hence, cGAMP-loaded VLPs have great potential as a vaccination platform. This suggests that the upstream signaling molecules of STING may participate in the process of innate immune resistance to viruses through the STING pathway. Most importantly, a timely optimum cGAS-STING response could provide protection against SARS-CoV-2 infection. The use of STING agonists such as diABZI exerts a beneficial effect by transiently stimulating IFN signaling triggered by cGAS-STING upon sensing SARS-CoV-2 infection. Therefore, a thorough understanding of the interrelationship between STING and SARS-CoV-2 is highly significant for designing treatment and preventive strategies.

## Author contributions

YW and MZ wrote the original manuscript and made the figure and the table. WLiu designed, supervised this review project, and revised the manuscript. ZM, CY, WLi, YZ, LS and JX provided guidance on correcting grammar and rhetoric. All authors contributed to the article and approved the submitted version.
